# Novel mutations in *SLC6A5* with benign course in hyperekplexia

**DOI:** 10.1101/mcs.a004465

**Published:** 2019-12

**Authors:** Hormos Salimi Dafsari, Amit Kawalia, Rosanne Sprute, Mert Karakaya, Anna Malenica, Peter Herkenrath, Peter Nürnberg, Susanne Motameny, Holger Thiele, Sebahattin Cirak

**Affiliations:** 1 Department of Pediatrics, Faculty of Medicine and University Hospital Cologne, University of Cologne, Cologne 50931, Germany;; 2 Center for Molecular Medicine (CMMC), Faculty of Medicine and University Hospital Cologne, University of Cologne, Cologne 50931, Germany;; 3 Cologne Center for Genomics (CCG), Faculty of Medicine, University of Cologne, Cologne 50931, Germany;; 4 Institute of Human Genetics, Faculty of Medicine and University Hospital Cologne, University of Cologne, Cologne 50931, Germany;; 5 Center for Rare Diseases, Faculty of Medicine and University Hospital Cologne, University of Cologne, Cologne 50931, Germany

**Keywords:** apneic episodes in infancy, equinovarus deformity, exaggerated startle response

## Abstract

Infants suffering from life-threatening apnea, stridor, cyanosis, and increased muscle tone may often be misdiagnosed with infantile seizures and inappropriately treated because of lack and delay in genetic diagnosis. Here, we report a patient with increased muscle tone after birth and hypertonic attacks with life-threatening apnea but no epileptiform patterns in EEG recordings. We identified novel compound heterozygous variants in *SLC6A5* (NM_004211.4:c.[1429T > C];[1430delC]) by trio whole-exome sequencing, containing a base deletion inherited by the asymptomatic mother leading to a frameshift (c.1430delC, p.Ser477PhefsTer9) and a de novo base exchange leading to an amino acid change (c.1429T > C, p.Ser477Pro). To date, there are four known disease-associated genes for primary hyperekplexia, all of which are involved in the functioning of glycinergic synapses. *SLC6A5* encodes the sodium- and chloride-dependent glycine transporter 2 (GlyT2), which recaptures glycine, a major inhibitory transmitter in the brainstem and spinal cord. The diagnosis altered the patient's medical care to his benefit because *SLC6A5* mutations with rather benign courses of hyperekplexia may be spared of needless pharmacotherapy. Symptoms eventually decreased in frequency until about once in 2 mo at 2 yr age. We present the first report of halting hyperekplexia episodes by maternal soothing in multiple instances. We highlight the importance of clarifying the genetic diagnosis by rapid next-generation sequencing techniques in this group of infantile apneic attacks with hyperekplexia due to the broad differential diagnoses.

## INTRODUCTION

Patients with sudden apneic attacks often present with diffuse patient histories and challenge pediatric physicians. Infant apnea may stem from anatomical obstructive or various central causes, oftentimes puzzling on-call physicians. Environmental or acquired etiologies of apneic attacks such as infections, premature birth, body temperature, sleep status, body position, and nicotine exposure play a major role in the differential diagnosis (Gao et al. 2017; Oishi et al. 2018). However, physicians are also oftentimes confronted with inborn genetic errors of the central nervous system—for example, congenital central hypoventilation with *PHO2XB* or *LBX1* mutations (Hernandez-Miranda et al. 2018; Zaidi et al. 2018) or congenital myasthenic syndromes with episodic apnea due to pathogenic variants in *CHAT* (Mallory et al. 2009).

Here, we present the clinical history and genetic investigation of a sporadic case affected with episodic apnea and stiffness immediately after birth resembling seizure-like episodes, and its clinical and genomic workup.

## RESULTS

### Clinical Presentation

We report an infantile case of episodic apnea and stiffness presented immediately after birth with seizure-like episodes, an increased muscle tone, and clubfoot positioning on both sides (left > right; HPO:0001762). The pregnancy was complicated with fetal seizure-like events in the last 2 mo of pregnancy as reported by the mother. The boy was born spontaneously at a gestational age of 41 + 5 wk with weight at 4355 g (90th percentile), length at 54 cm (60th percentile), and head circumference at 35.5 cm (30th percentile) from non-consanguineous German parents (APGAR 1/10/10, umbilical artery pH 7.16). There was no family history of relevant neurological diseases.

The patient was admitted to our hospital 7 d after birth because of a sudden apneic attack. In the emergency department, the patient showed attacks with increased muscle tone in the lower jaw, jitter, irritability, and tachycardia (170 bpm). This episode was initially interpreted as a seizure by on-call clinicians. During the setup of an intravenous line, he presented myoclonic jerks of all extremities for 5 sec. The seizure-like attacks ceased on intravenous administration of lorazepam. There were no evidence or anamnestic hints for an infection or other acquired or environmental etiology for the attacks despite investigations.

Again, on the second day of hospitalization we observed attacks that mimicked generalized seizures and seized on the administration of phenobarbital. An EEG examination showed no relevant findings with an ordinary baseline activity without epileptiform patterns or foci.

We started levetiracetam in a dose of 40 mg/kg/d (85 mg–0–85 mg), which improved the initially increased muscle tone. A cranial magnetic resonance imaging examination 2 d after admission revealed a non-space-consuming subdural hematoma most likely due to birth trauma. A follow-up transfontanellar sonography after a few weeks showed no relevant findings, which mostly occurs with resorption in normal sonographic brain texture (Bowerman et al. 1984). To exclude an underlying epilepsy syndrome, we performed EEG examinations on five different occasions throughout hospitalization as well as 3 mo later. Repeatedly, none of the EEGs showed epileptiform patterns or foci. Newborn metabolic blood screening according to the German national guidelines was unremarkable (Lindner et al. 2011). Subsequent CSF workup during hospitalization for a GLUT1 or neurotransmitter defect were also unremarkable.

On discharge, we continued levetiracetam (85 mg–0–85 mg). For 4 mo, the tonic-jittery attacks appeared daily, mainly in a prone position. At the age of 6 mo, the frequency of attacks decreased to once in every 2 or 3 wk, eventually once in every 1 or 2 mo. We refer to the video of two instances of hyperekplexia attacks at the age of 2 mo in our Supplemental Material (Supplemental Video S1). At toddler age, the tonic-jittery attacks were mostly produced by aural stimuli. Eventually, we discontinued the anti-epileptic medication at the age of 3 yr. During attacks, the attacks ceased on maternal soothing. Further outpatient follow-up in our neuropediatric clinic showed unremarkable alimentary and neurological development according to age. During the most recent follow-up in 2018 at the age of 4 yr, the patient had an average formal IQ of 100 according to Kaufman Assessment Battery for Children (ABC-II). We observed no further apneic attacks and the startle reactions only appear once in every 2 mo. Of note, the attacks only appeared when the child was lying prone; however, when lying supine the boy did not react to nose tapping and acoustic or tactile stimuli. The attacks were occurring at a frequency of once or twice a year and were induced especially by aural stimuli (Supplemental Video S1). In neurological follow-up examinations, we have not observed any pathological reflexes, delay of motor development, nor radiological signs in transfontanellar sonography that would suggest a beginning cerebral palsy. Of note, clubfeet may be a sign of an increased muscle tone (Karakaya et al. 2016). In our patient, the clubfeet were treated with an orthopedic redression in plaster and left no remaining defect on feet posture or gait.

### Genomic Analyses

**Figure 1.**
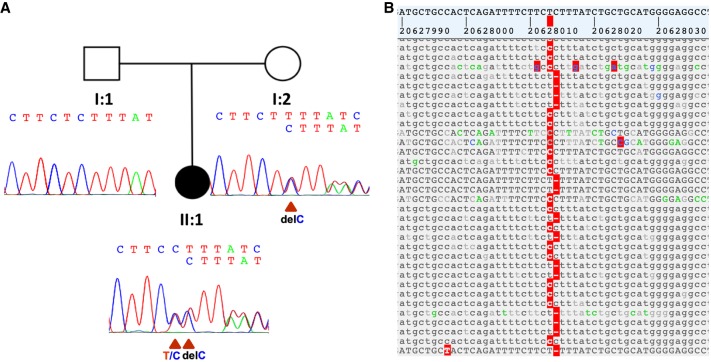
(*A*) Pedigree and chromatogram of the two variants in *SLC6A5*. The mother is a heterozygous carrier of a deletion (c.1430delC, p.Ser477PhefsTer9). The patient carries the deletion inherited by the mother and additionally showed another heterozygous de novo mutation (c.1429T > C, p.Ser477Pro). (*B*) Review of alignments in patient's compound heterozygous variant from whole-exome sequencing (WES) with the two mutations located next to each other (varbank; https://varbank.ccg.uni-koeln.de).

To uncover the genetic cause of the attacks, we first performed Mendeliome sequencing (see Methods and Supplemental Table S4; Fazeli et al. 2016). After initial filtering, we had only observed one variant in *SLC6A5* and initially no other conclusive results that could have explained the clinical features sufficiently. In search for the genetic diagnosis, we performed trio whole-exome sequencing (WES) (see Methods and Supplemental Tables S5–S7), which revealed a compound heterozygous variant in *SLC6A5* (NM_004211.4:c.[1429T > C];[1430delC]), consisting of a deletion inherited by the mother (c.1430delC, p.Ser477PhefsTer9) and a (likely) de novo base exchange (c.1429T > C, p.Ser477Pro) in the patient (see Fig. 1). The patient has a novel variant, which was previously not found in ClinVar or gnomAD. However, the mother's variant has a reported frequency of 2/246158 in gnomAD. Both variants have been listed now in ClinVar (SCV000897641 and SCV000897642). The bioinformatic reanalysis and annotation of the Mendeliome next-generation sequencing data confirmed the results as well, which was found by the trio WES results in *SLC6A5* as shown in Supplemental Tables S5–S7.

**Figure 2.**
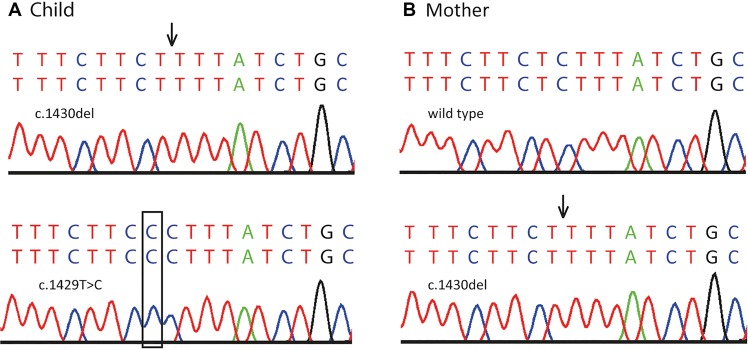
Dideoxy sequencing results after subcloning with TOPO TA cloning kit (see Methods). (*A*) The *upper* row shows confirmation of the deletion (c.1430delC, p. Ser477FfsTer9; black arrow); the *lower* row shows the de novo mutation (c.1429T > C, p. Ser477Pro; black box) in the index patient. (*B*) The *upper* row shows wild-type sequence; the *lower* row shows confirmation of the carrier status of a deletion (c.1430delC, p. Ser477FfsTer9; black arrow) in the mother of the index patient.

To double-check the sequences and the phasing, we subcloned PCR products of the peripheral blood DNA of the index patient and his mother in order to split the *SLC6A5* alleles into separate plasmids. We used TOPO-TA cloning and subsequently performed a plasmid purification and dideoxy sequencing of the alleles in the plasmid separately (see Methods). The dideoxy sequencing results confirmed these variants in the patient and his mother (see Fig. 2). We also performed kinship analysis to confirm that the patient is indeed the biological offspring of these parents (see Supplemental Methods; Supplemental Table S3).

**Figure 3.**
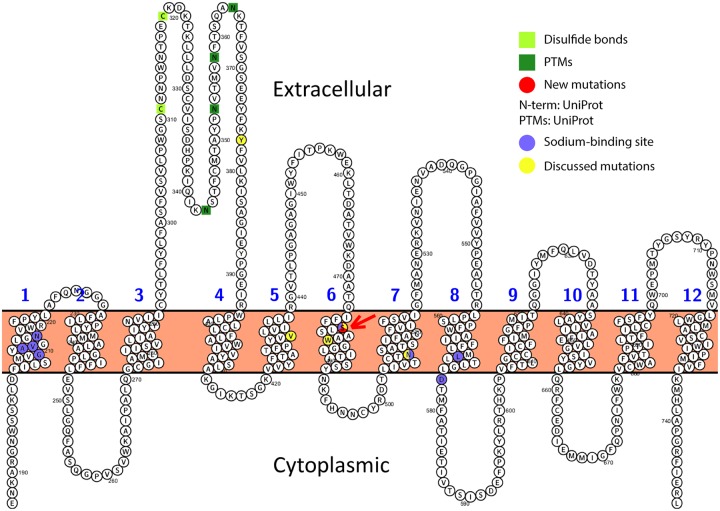
Visualization of SLC6A5 as a transmembrane protein with 12 transmembrane regions, and the mutation in our patient on position p. Ser477 in region 6 is marked in red with a red arrow, as well as the missense mutations (p.Trp482Arg, p.Asn509Ser), nonsense mutation (p.Tyr377Ter), and frameshift mutation (p.Val432PhefsTer99) (Rees et al. 2001; Benito-Muñoz et al. 2018). Position 477 is known as a Na^+^-binding site. Each amino acid is abbreviated by its first-letter code.

*SLC6A5* encodes a sodium- and chloride-dependent glycine neurotransmitter transporter (GlyT2) consisting of 12 transmembrane regions. The mutations on position p.Ser477 are located in transmembrane region 6 (see Fig. 3), which has a role in transporter binding and transporter activity. Position p.Ser477 is expected to serve as one of eight sodium-binding sites by similarity to DAT (sodium-dependent dopamine transporter) according to a previously published analysis (Benito-Muñoz et al. 2018).

To elaborate on the importance of the residue p.Ser477 for metal binding, multiple sequence alignment was performed for SLC6A5 between the sodium-dependent dopamine transporter from *Drosophila melanogaster* (DAT_DROME) as well as members of the human SLC6 family of sodium- and chloride-dependent neurotransmitter transporters (see Supplemental Fig. S1). The alignment was generated using the NCBI HomoloGene Protein Multiple Alignment platform, analyzed with the MUSCLE algorithm (Edgar 2004), provided by the Jalview web service, and visualized with the Jalview online tool (version 2.11.0) (Waterhouse et al. 2009). The secondary structure was predicted with the JPred Secondary Structure Prediction tool (Drozdetskiy and Cole 2015). Amino acid color labels were selected for the block substitution matrix 62.

In addition, a copy-number variant (CNV) analysis was performed to ensure we have not overlooked any other pathogenic variant. However, none of the detected CNVs shows any relevance to the phenotype (see Supplemental Tables S8–S10).

## DISCUSSION

We report a 2-yr-old boy from non-consanguineous German parents with tonic-jittery attacks with tachycardia and intermittently aggravated hypertonic phases in extremities that led to life-threatening apnea with cyanosis immediately after birth. In deciphering the underlying etiology, we identified novel compound heterozygous variants in *SLC6A5* (NM_004211.4:c.[1429T > C];[1430delC]), containing a deletion inherited by the asymptomatic mother (c.1430delC, p.Ser477PhefsTer9) and a likely de novo base exchange (c.1429T > C, p.Ser477Pro). A plausible alternative mode of inheritance may be germline mosaicism of the patient's father.

Congenital hyperekplexia is a rare, potentially treatable neurogenetic disorder, and the diagnosis has been largely based on clinical findings (Thomas 2015). Affected individuals typically show generalized stiffness immediately or soon after birth. An excessive startle reflex to unexpected stimuli is the second main feature in hyperekplexia. Startle episodes are reported in some cases before birth (Thomas 2015). Although the symptoms are clearly defined, hyperekplexia can be confused with neonatal epilepsy, thus delaying diagnosis. Patients with hyperekplexia stay conscious during the tonic-jittery attacks, which distinguishes the disease from epileptic seizures. Although the symptoms often diminish during the first years of life, the excessive startle response can persist well into adulthood, leading to serious injuries from unprotected falls (Bode and Lynch 2014).

Although no epileptiform discharges were observed, the patient was treated with anti-epileptic medication (levetiracetam) for 6 mo because the use of levetiracetam in a child with hyperekplexia was reported to have dramatically decreased the frequency of attacks (Hussain et al. 2013). After the introduction of levetiracetam in our patient, we have observed a relaxation of initially increased muscle tone, but there were no changes in the hyperekplexia attacks. Thus, we suggest that a rather benign course of hyperekplexia with *SLC6A5* mutations may render a continuous pharmacotherapeutic management dispensable. However, it should be debated on the basis of the individual case if the patient may benefit from a preventive pharmacotherapy in the first 3 mo of life because of severe apnea attacks or a *pro re nata* (PRN) medication (e.g., buccal midazolam). Of note, we present the first report of halting hyperekplexia attacks with maternal soothing and avoiding surrounding auditory stimuli.

To this date, we have knowledge of four disease-causing genes in primary hyperekplexia: glycine receptor subunit α 1 (*GLRA1*; OMIM #138491) (Shiang et al. 1993), glycine receptor subunit β (*GLRB*; OMIM #138492) (Grenningloh et al. 1990), *ATAD1* (ATPase family, AAA domain-containing, member 1, OMIM #614452) (Ahrens-Nicklas et al. 2017), and *SLC6A5,* which encodes for the GlyT2 glycine transporter (Mine et al. 2015). All four genes affect major constituent parts of the inhibitory glycinergic system (Harvey et al. 2008). *ARHGEF9*, encoding collibistin, and *GPHN*, encoding gephyrin, were also candidates but are now regarded as atypical cases and undiagnosed clinical mimics of early neonatal hypertonia and excessive startle (Chung et al. 2013).

In GlyT2-KO mice, glycinergic inhibition, such as in hyperekplexia, has proven to be mainly relevant in feedback regulation of respiratory reflexes. GlyT2 inhibition leads to depletion of intracellular glycine storage and limitation of glycine accumulation in synaptic vesicles (Morrow et al. 1998). In recurring cases without clinical clues, genetic workup might be useful to shed some light on the nature and course of the disease. For instance, if patients present with startle reflexes, it may allude to a rather benign variant such as hyperekplexia. A forced flexion of head and legs over the trunk (“Vigevano maneuver”) has been reported to stop sudden attacks of hyperekplexia in infancy (Vigevano et al. 1989). Therapeutic management in hyperekplexia might include medication with an allosteric potentiator of the inhibitory GABA_A_ receptor clonazepam in patients with variants in *GLRA1* (Tijssen et al. 1997) and *SLC6A5* (Bakker et al. 2009; Thomas 2015). The stimulation of P2X purinergic receptors with βγ-methylene adenosine 5′-triphosphate has also been shown to induce the up-regulation of GlyT2 transport activity by increasing total and plasma membrane expression and reducing transporter ubiquitination (Villarejo-López et al. 2017). As a nonharmful therapeutic strategy in our case, maternal soothing was observed as halting hyperekplexia attacks in repeated instances.

We report two variants, which are located directly next to each other (see Fig. 1), and confirmed them by subcloning and subsequent plasmid sequencing.

Our results confirm that the healthy mother is a carrier of a deletion on position c.1430delC—a heterozygous frameshift variant that has been predicted to be subject to nonsense-mediated decay (NMD) by the NMDEsc Predictor. Because the mother remains asymptomatic, we may argue that the deficient SLC6A5 is partially cleared away by the NMD pathway or does not cause any other dominant negative effect. In a previous report, asymptomatic parents of multiple patients with *SLC6A5*-related hyperekplexia have been observed with truncating variants (Rees et al. 2006), which is in line with our observation.

In addition to this frameshift variant, the patient had a missense mutation (p.Ser477Pro) with a change from serine to proline. Please see Figure 3 for a comparison of our patient's mutation site as well as the missense, nonsense, and frameshift mutations that are discussed in the following. In a previous report of a severely affected individual (Rees et al. 2006), confocal microscopy of transfected HEK293 cells showed that nonmutated EGFP-hGlyT2 was readily expressed at the cell surface, whereas the mutants (p.Tyr377Ter and p.Val432PhefsTer99 among others) appeared to be cytoplasmic and showed no distinct expression at the cell surface with reduced [^3^H]glycine uptake. Furthermore, Rees and colleagues have also shown in two-electrode voltage clamp analysis in *Xenopus* oocytes that a mutation in p.Trp482Arg did not respond to glycine (up to 10 mM), but was present at the cell surface as demonstrated by sodium-dependent and glycine-insensitive transient currents (see Fig. 3)—that is, the mutants p.Trp482Arg (transmembrane region 6) were functionally inert. In another report on the sodium-binding activity in SLC6A5, the mutational site in our patient, p.Ser477 in transmembrane region 6, was observed to serve as one of eight sodium-binding sites based on an homology model of GlyT2 *d*DAT (sodium-dependent dopamine transporter, Q7K4Y6, DAT_DROME) and was confirmed experimentally by electrophysiologcal examinations (Benito-Muñoz et al. 2018). In Supplemental Figure S1, we show a multiple sequence alignment of human SLC6A5 between the sodium-dependent dopamine transporter from *Drosophila melanogaster* (DAT_DROME) and paralog members of the human SLC6 family. This alignment indicates that the residue p.Ser477 is strictly conserved through sodium- and chloride-dependent neurotransmitter transporters, highlighting its importance in sodium binding. Overall secondary structure prediction (jnetpred) demonstrates the location of p.Ser477 in an α-helical section, consistent with its transmembrane location. As expected, no coiled-coil structure was predicted for this section (Lupas et al. 1991). Prediction of solvent accessibility (Jnet Burial) shows a medium exposure of residue p.Ser477. Thus, we conjecture that the missense variant in our patient (c.1429C > T, p.Ser477Pro) serves as a pathogenic mutation on the protein level because of the strict conservation throughout species, the damaging effect of a proline introduction in the α-helix, and the aforementioned electrophysiological observations of deficient sodium binding (Benito-Muñoz et al. 2018). On the basis of these findings and the experimental observations by Rees and colleagues, we hypothesize that the missense mutation (p.Ser477Pro) may lead to deficient metal binding in GlyT2 at transmembrane region 6, which renders it functionally inert. and to reduced expression levels because of the disrupting effect of proline onto the α-helix.

The patient's life-threatening apneic attacks first raised suspicions toward congenital hypoventilation as a severe differential diagnosis to hyperekplexia. *LBX1* and *PHOX2B* mutations have previously been shown to impair the development of a small subpopulation of neurons in the medulla oblongata that are essential for respiratory control (Hernandez-Miranda et al. 2018). Other differential diagnoses of neuromuscular symptoms with an infantile onset may stem from “channelopathies” (i.e., a heterogeneous group of disorders resulting from the dysfunction of transmembrane ion channels). These more severe cases comprise patients with mutations in genes that are components of the nonselective sodium leak channel complex (NALCN channelosome) and—depending on the inheritance pattern—either present with muscular hypertonia and distal contractures (Karakaya et al. 2016) or hypotonia, psychomotor retardation, and dysmorphic features (Bramswig et al. 2018), as well as patients with mutations in the sodium voltage-gated channel α subunit 4 gene (*SCN4A*), which may present with congenital myopathy or as congenital myasthenic syndrome (Sloth et al. 2018; Elia et al. 2019).

Hyperekplexia patients with a benign phenotype and variants in *SLC6A5* are significantly less likely to have recurrent infantile apnea than those with *GLRA1* variants (Thomas 2015). Moreover, patients with variants in *GLRB* and *SLC6A5* are more likely to have a developmental delay than those with *GLRA1* variants. Thus, an early genetic workup helps in recognizing the patient's symptoms, providing parents with genetic counseling, and avoiding unnecessary medication and its accompanying adverse effects in early postnatal development. Next-generation sequencing (NGS) to uncover the underlying cause is indicated because of the broad spectrum of genetic differential diagnosis for apneic attacks and epilepsy-like clinical presentations.

Diagnosing rare diseases with NGS in perinatal settings has become highly rapid, economical, and efficient, but it comes with careful consideration of parental consent, ethical framework, and sparing trauma for patients and parents (Daoud et al. 2016; Fazeli et al. 2016; Poulsen et al. 2016; Borghesi et al. 2017; Kuehne et al. 2019). Importantly, receiving a genetic diagnosis might enable physicians to administer specific therapy or at least deter unnecessary drug exposure.

In conclusion, we report a novel compound heterozygous variant in *SLC6A5* with already well-established symptoms that may have been overlooked initially because of the broad differential diagnoses of apneic attacks. If—as in this case—a variant in *SLC6A5* is revealed to cause the disease, the patient is expected to have a benign form of hyperekplexia. We report the first instances of stopping hyperekplexia attacks with maternal soothing, thus the patient could be spared anti-epileptic medication. Because the patients are conscious during attacks, the family members could be informed of strategies to halt the attacks without using medication, such as soothing the patient. Lastly, we may offer genetic counseling to the patient's family regarding the expected ordinary thriving and sensomotoric development in this benign disease course.

## METHODS

Written informed consent was obtained from the parents for genetic investigations and recording and publishing of the disease-related information. The study was approved by the institutional review board of the Ethics Committee of the University Hospital of Cologne.

To uncover the genetic cause in this family, we performed Mendeliome sequencing, a commercial gene panel (Illumina TruSight One, Illumina) including 4.813 genes responsible for rare diseases (see Supplemental Tables S1 and S4; Fazeli et al. 2016; Alawbathani et al. 2018). The sequencing was performed on a MiSeq sequencer (Illumina) using the TruSight One chemistry for target extraction (Illumina). Because it was inconclusive in the first analysis, we performed trio whole-exome sequencing, which led to the diagnosis. Reanalysis of the Mendeliome confirmed the results. Genomic DNA samples isolated from peripheral blood of the index patient and his parents were enriched with the NimbleGen SeqCap EZ Human Exome Library v2.0 (Roche) following the manufacturer's instructions. The trio was sequenced on a HiSeq 2000 sequencer (Illumina) with 2 × 101-bp reads, producing a mean coverage of the target regions of 94× for the index patient, 89× for the father, and 147× for the mother (see Supplemental Tables S1 and S5–S7). To confirm the variants in *SLC6A5* and to validate the cosegregation within the family, we performed dideoxy sequencing.

Using early versions of the Cologne Center for Genomics exome pipeline, the sequencing data of the Mendeliome sequencing was analyzed with version 2.10, and the WES trio and the reanalysis of Mendeliome sequencing data were analyzed with version 2.14, only with differences in technical fixes—for example, activating Ion Torrent and Illumina gene panels, disabling downsampling in variant callers, and various bug fixes in parameter parsing or disk space usage (Kawalia et al. 2015). For further bioinformatics analysis of NGS data, refer to the Methods section in our Supplemental Material.

The variants were filtered for a de novo and compound heterozygous inheritance model without consanguine familiar background; with an allele read frequency window of 25%–75%. Variants were considered with a minor allele frequency of 0.1% or less. From a total number of 16 rare functional variants (see Supplemental Table S2), we checked the variants for a quality of >100, nonsynonymous coding, polymorphism predictions. We classified the remaining variants according to the American College of Medical Genetics and Genomics–Association for Molecular Pathology (ACMG–AMP) guidelines and refined Sherloc criteria (Richards et al. 2015; Nykamp et al. 2017). Solely the mentioned variants in *SLC6A5* fitted to all of the filter criteria, were classified as pathogenic in both variant classifications, and could be matched to the phenotype of our patient. Table 1 shows the gene variants, according to the ACMG–AMP criteria, and refined Sherloc criteria (Nykamp et al. 2017) in the remaining variants, including four compound-heterozygous variants of uncertain significance in the ATP Binding Cassette Subfamily C Member 6 (*ABCC6)* gene and *KIAA0513* gene, and both *SLC6A5* variants we present here, which are highlighted in Supplemental Table S11. To evaluate if a variant was subjected to NMD, we used the NMDEsc Predictor online tool (https://nmdprediction.shinyapps.io/nmdescpredictor/). In addition to the above-mentioned resources, the variants were also checked in gnomAD (https://gnomad.broadinstitute.org/) for reports in exome- or genome-wide population studies and ClinVar (https://www.ncbi.nlm.nih.gov/clinvar/) for supporting evidence and clinical significance.

**Table 1.** Rare coding variants in the compound heterozygous state detected in the index patient by trio WES according to guidelines of the ACMG–AMP and refined Sherloc criteria

Gene	Transcript	Variants	MAF	ACMG–AMP scoring	Sherloc scoring	ACMG–AMP and Sherloc classification	Inheritance	ClinVar	gnomAD	Associated phenotype
*ABCC6*	NM_001171.5	c.3089G > A; p.Arg1030Gln	0.000016 (ExAC) 0 (GME)	PM2, PM5, PP2, PP3, BP5	P1.5 B1	Uncertain significance	From mother	n/a	6 Heterozygous	#264800 Pseudoxanthoma elasticum
*ABCC6*	NM_001171.5	c.2359G > A; p.Val787Ile	0.000565 (ExAC) 0.00352 (GME)	PS1, PP2, PP3, BP5, BS1	P2.5 B3		From father	419855	163 Heterozygous	
*KIAA0513*	NM_014732.2	c.727G > C; p.Gly243Arg	0.000008 (ExAC) 0 (GME)	PM2, PP3, BP5	P1.5 B1	Uncertain significance	From father	n/a	n/a	n/a
*KIAA0513*	NM_014732.2	c.855G > T; p.Lys285Asn	0 (ExAC) 0 (GME)	PM2, BP4, BP5	P1 B1		From mother	n/a	n/a	
*SLC6A5*	NM_004211.4	c.1429T > C; p.Ser477Pro	0.000016 (ExAC) 0 (GME)	PS2, PM1, PM2, PM3, PP2, PP3, PP4	P14.5	Pathogenic	Likely de novo	897642	n/a	#614618 Hyperekplexia 3
*SLC6A5*	NM_004211.4	c.1430delC; p.Ser477PhefsTer9	0 (ExAC) 0 (GME)	PVS1, PM1, PM2, PM3, PP2, PP3, PP4	P13.5		From mother	897641	2 Heterozygous	

Information in the table has been taken from Richards et al. 2015; Nykamp et al. 2017.

The ACMG and Sherloc scores were called for each variant, the ACMG–AMP and Sherloc classification was called together for compound heterozygous variants. Only the *SLC6A5* variant was clearly classified as pathogenic. The variants in *ABCC6* locate to the nucleotide-binding fold 1 (p.Val787Ile) and the seventh cytoplasmic loop (p.Arg1030Gln), whereas p.Val787Ile has been published in a patient with Pseudoxanthoma elasticum (PXE) leading to strokes due to vascular mineralization. However, ACMG criteria imply uncertain significance for variants and there were no other neurological manifestations, which renders the *ABCC6* variants highly unlikely to be the primary cause of the patient's disease. Variants in *KIAA0513* have been associated with schizophrenia; however, as they were not reported in combination with seizure-like episodes, a modifying role cannot be excluded. For *SLC6A5,* the identified homozygous frameshift variant explains the phenotype of hyperekplexia adequately.

(ACMG–AMP) American College of Medical Genetics and Genomics–Association for Molecular Pathology, (WES) whole-exome sequencing, (MAF) minor allele frequency, (ExAC) Exome Aggregation Consortium, (GME) Greater Middle East Variome Project, (ClinVar ID) variation ID from ClinVar platform, (gnomAD) Genome Aggregation Database, (n/a) not available.

To confirm the relations of patient and parents, we also performed kinship analysis with varbank by analyzing the proportion of shared rare alleles (see Supplemental Methods and Supplemental Table S3 for further details).

The pipeline performed CNV calling for the patient, father, and mother individually, using three different callers: ExomeDepth (Plagnol et al. 2012), XHMM (Fromer et al. 2012), and CoNIFER (Krumm et al. 2012). For more details on CNV calling, please see Supplemental Tables S8–S10 for called de novo CNVs and details about callers.

In a next step, we wanted to confirm the allele specificity of the *SLC6A5* variants. After a standard PCR procedure to child and mother's samples with HotStar HiFidelity DNA polymerase (QIAGEN) with a proofreading 3′ to 5′ exonuclease activity, the PCR products were purified to remove proofreading enyzme with the PCR clean-up Gel extraction kit (Macherey-Nagel) according to the protocol.

After purification of the PCR product, a 3′ A-overhang was added by incubation of the PCR product with a nonproofreading MyTaq DNA polymerase, dNTPs, and MyTaq 1× PCR buffer (BIOLINE) for 10 min at 72°C. The product was ligated into the pCR4-TOPO TA Vector (Invitrogen) according to the manufacturer's protocol for the “TOPO TA Cloning Kit for sequencing.” The ligation product was transformed into “One Shot Mach1 Phage-Resistant” Chemically Competent *E. coli* (Thermo Fisher Scientific). Cells were plated and incubated overnight at 37°C. Several colonies were picked to inoculate 5-mL cultures and incubated overnight at 37°C. Plasmids were purified with the NucleoSpin Plasmid kit (Machery-Nagel). Subsequent dideoxy sequencing of the plasmids insert confirmed the above-mentioned variants in the index patient and his mother (see Fig. 2).

## ADDITIONAL INFORMATION

### Data Deposition and Access

The pathogenic *SLC6A5* variants have been submitted to ClinVar (http://www.ncbi.nlm.nih.gov/clinvar/) and can be found under accession numbers SCV000897641 and SCV000897642. We have no further data to be deposited, because we are not allowed to publish the full exome variant data set based on IRB approval and patient consent.

### Ethics Statement

Informed consent was obtained from the patient and parents for genetic investigations and recording and publishing of the disease-related information. The study was approved by the institutional review board of the Ethics Committee of the University Hospital of Cologne.

### Acknowledgments

We thank Kerstin Becker for helping with the figures in this manuscript. This work was supported by the Deutsche Forschungsgemeinschaft Emmy Noether Grant (CI 218/1-1) to S.C. and Muscular Dystrophy Association (Developmental Grant Cirak). H.S.D. was supported by the Gerok program of the Faculty of Medicine, University of Cologne. We also thank the Regional Computing Center of the University of Cologne (RRZK) for providing computing time for the bioinformatics analyses on the DFG-funded High Performance Computing (HPC) system CHEOPS, as well as for support.

### Author Contributions

H.S.D. analyzed clinical, genetic, and bioinformatic data and wrote the manuscript. A.M. summarized clinical findings. P.H. contributed to the clinical diagnosis, description, and management of the patient. M.K. contributed to the genetic workup and data analysis and revised the manuscript. A.K., R.S., A.M., P.N., S.M., and H.T. contributed the genetic and bioinformatic analysis of the data and revised the manuscript. S.C. obtained funding, analyzed the data, coordinated and supervised the work, and revised the manuscript. All authors approved the manuscript before submission.

### Competing Interest Statement

The authors have declared no competing interest.

### Referees

Gholson Lyon

Anonymous

## Supplementary Material

Supplemental Material
