# Supplementary material for: Novel mutations in SLC6A5 with benign course in hyperekplexia
Source: Cold Spring Harb Mol Case Stud. 2019 Dec;5(6):a004465. doi: 10.1101/mcs.a004465 (PMC6913151; doi:10.1101/mcs.a004465)
Supplement: Supplemental Material [file supp_5_6_a004465__index.html]

Novel mutations in SLC6A5 with benign course in hyperekplexia — Supplemental Material 

# Novel mutations in *SLC6A5* with benign course in hyperekplexia

## Supplemental Material

- Supplemental\_Material\_Figure\_Tables.docx
- Supplemental\_Table\_S4\_Hyperekplexia\_SLC6A5\_Patient\_MendelianPanel\_Coverage.xlsx
- Supplemental\_Table\_S5\_Hyperekplexia\_SLC6A5\_Mother\_Exome\_Coverage.xlsx
- Supplemental\_Table\_S6\_Hyperekplexia\_SLC6A5\_Father\_Exome\_Coverage.xlsx
- Supplemental\_Table\_S7\_Hyperekplexia\_SLC6A5\_Patient\_Exome\_Coverage.xlsx
- Supplemental\_Table\_S8\_Hyperekplexia\_SLC6A5.conifer.bed.denovo.xlsx
- Supplemental\_Table\_S9\_Hyperekplexia\_SLC6A5.xhmm.DATA.xcnv.denovo.xlsx
- Supplemental\_Table\_S10\_Hyperekplexia\_SLC6A5.exomedepth.tab.denovo.xlsx
- Supplemental\_Video\_S1.mov
