# Supplementary material for: Novel mutations in SLC6A5 with benign course in hyperekplexia
Source: Cold Spring Harb Mol Case Stud. 2019 Dec;5(6):a004465. doi: 10.1101/mcs.a004465 (PMC6913151; doi:10.1101/mcs.a004465)
Supplement: Supplemental Material [file supp_mcs.a004465_Supplemental_Material_Figure_Tables.docx]

**Supplementary Material:**

**Novel Mutations in *SLC6A5* with benign course in hyperekplexia**

**SUPPLEMENTARY METHODS**

Sequence reads from the trio were aligned to the GRCh37 human genome sequence using bwa-aln (version 0.6.2) (Li and Durbin 2009). Duplicates were marked using Picard (version 1.64) (http://broadinstitute.github.io/picard/) followed by base quality score recalibration and local indel realignment using GATK (version 1.6.11) (McKenna et al. 2010). Variants were called in the individual trio members using samtools mpileup (version 0.1.18) (Li et al. 2009) and GATK UnifiedGenotyper (version 1.6.11). On the variants called by GATK UnifiedGenotyper, variant quality score recalibration using GATK (version 1.6.11) was performed. Since we needed to identify the relevant inheritance type, we also checked for *de-novo* variants in the child, which were called using deNovoGear (version 0.5.1) (Ramu et al. 2013). All variants from the different callers were merged for each trio member and annotated using inhouse-developed scripts. These scripts utilize the information from various public databases (dbSNP (Sherry et al. 2001), 1000 Genomes Project (Abecasis et al. 2012), Exome Variant Server (EVS, http://evs.gs.washington.edu/EVS/), dbVAR and DGVa (Lappalainen et al. 2013), GERP (Davydov et al. 2010), ENSEMBL (Flicek et al. 2014), commercial HGMD professional database (Stenson et al. 2009)) and tools (POLYPHEN (Adzhubei et al. 2010), SIFT (Kumar et al. 2009), RVIS (Petrovski et al. 2013)) and also contain a splice site analysis (Yeo and Burge 2004). The annotated variant lists were uploaded to the Cologne Center for Genomic’s Varbank (https://varbank.ccg.uni-koeln.de) database for variant filtering. Using early versions of the Cologne Center for Genomics exome pipeline, the sequencing data of the Mendeliome sequencing was analyzed with version 2.10, and the WES trio and the re-analysis of Mendeliome data were analyzed with version 2.14, only with differences in technical fixes, e.g., activating Iontorrent and Illumina gene panels, disabling down-sampling in variant callers, and various bugfixes in parameter parsing or disk space usage (Kawalia et al. 2015).

**CNV callers**

As mentioned in method section, we have used three different callers: ExomeDepth, XHMM and CoNIFER. All of these three callers use read-depth approach, which fundamentally measure the unusual divergence in read depth in comparison to the normal range in order to discover mainly deletion (reduced read depth) or duplication (higher read depth) (Alkan et al. 2011; Escaramis et al. 2015). However, they use different methods for coverage normalization and CNV identification. XHMM uses principal component analysis (PCA) and hidden markov model (HMM), whereas CoNIFER uses singular value decomposition (SVD) method for systematic biases correction and CNV detection. ExomeDepth uses slightly different approach, i.e. beta-binomial model, to build a baseline for coverage in order to call CNVs. A detailed comparison of these tools has previously been put forward (Tan et al. 2014; Hong et al. 2016).

CNV calling was performed for the patient, father and mother individually using the aforementioned three CNV callers. To avoid coverage bias during calling (Fromer et al. 2012; Krumm et al. 2012), each of the samples were compared against a reference set of 75 samples of mixed phenotypes sequenced with the same enrichment kit (Nimblegen v2). For the sex chromosomes, sex-matched reference sets were used (40 female / 38 male). In order to uncover potential de-novo events, the overlap between the patient’s CNV calls and the parent’s CNV calls was computed (see Supplementary Tables S8-10).

**Kinship analyses**

We performed kinship analysis by analysing the proportion of shared rare alleles. For these kinship analyses, we used Varbank (https://varbank.ccg.uni-koeln.de/) between samples from index patient, father and mother (see Supplementary Table S3). The filters for selecting autosomal SNPs are: known SNP with minor allele frequency <0.001; target distance <100 bp; passed GATKs variant quality score recalibration (VQSR) filter. In theory, the proposed sharing of rare marker alleles is around 50% for relationships between children and parents. With the method here, the values can accordingly be lower. The analysis table shows a comparison of every selected sample against each other. The sharing between two individuals is measured in both directions.

**Supplementary Table S1:**

|  | **Patient**  **(Mendeliome)** | **Patient (Exome)** | **Father** | **Mother** |
| --- | --- | --- | --- | --- |
| **Read length** | 151 | 101 | 101 | 101 |
| **Mean Coverage** | 85 | 94 | 89 | 147 |
| **Coverage 2x** | 98.9 | 98.8 | 98.7 | 98.8 |
| **Coverage 10x** | 97.3 | 97.5 | 97 | 98.1 |
| **Coverage 20x** | 93.7 | 94.1 | 92.6 | 96.4 |
| **Coverage 30x** | 87.9 | 88.7 | 85.9 | 93.8 |
| **Total reads** | 15537048 | 76340294 | 70190716 | 120140770 |
| **Unique reads (UR)** | 13248816 | 71144094 | 66170824 | 110460690 |
| **Unique mapped reads (UMR)** | 12582324 | 66511255 | 61933030 | 103467476 |
| **%UR** | 85.3 | 93.2 | 94.3 | 91.9 |
| **%UMR** | 95.0 | 93.5 | 93.6 | 93.7 |

**Supplementary Table S1:** Sequencing coverage table for the patient with Mendeliome sequencing and for the patient, father, and mother with trio Whole Exome sequencing (WES). Read length, mean coverage, coverage 2x, coverage 10x, coverage 20x, coverage 30x, total reads, unique reads (UR), unique mapped reads (UMR), and the percentages of UR and UMR relative to total reads are listed for each sequencing run in this table.

**Supplementary Table S2:**

|  | ROH:60 |
| --- | --- |
| Rare functional variants (RFV) | 16 |
| RFVs in good sequence quality (Q>100) | 11 |
| Non-synonymous coding, indels  (with intronic mutations close to splice site <20 bp up/downstream) | 11 |
| Non-polymorphism predictions | 9 |
| RFVs related to movement disorders | 1 |

**Supplementary Table S2:** Results of the variant filtering and the specific criteria we applied on the dataset. With an ROH of 60, we filtered out 16 rare functional variants (RFV). 11 RFV hat a sequence quality above 100 and were classified as non-synonymous coding or indels, including intronic mutations close to splice site <20bp up-/downstream. Of these remaining variants, nine variants were predicted as non-polymorphisms and only one variant was related to movement disorders (*SLC6A5*).

**Supplementary Table S3:**

| compared samples | number of shared rare alleles | number of rare alleles from sample 1 | number of rare alleles from sample 2 | percentage of allele sharing sample1/sample2 | percentage of allele sharing sample2/sample1 |
| --- | --- | --- | --- | --- | --- |
| sample 1: patient  sample 2: father | 79 | 210 | 187 | 38 | 42 |
| sample 1: patient  sample 2: mother | 111 | 210 | 243 | 53 | 46 |
| sample 1: mother  sample 2: father | 0 | 243 | 187 | 0 | 0 |

**Supplementary Table S3:** Kinship analysis according to Varbank (https://varbank.ccg.uni-koeln.de/) between samples compared for shared rare alleles. The sharing between individual is measured in both directions. The patient shares 38% of rare alleles with his father, or 42% vice versa respectively. The patient shares 53% of rare alleles with his mother, or 46% vice versa respectively. See Methods in Supplementary Material for further details.

**Supplementary Tables S4-7**

| **Gene** | **Chromosome** | **HGVS DNA Reference** | **HGVS Protein Reference** | **Variant Type** | **Predicted Effect** | **dbSNP/dbVar ID** | **Genotype** | **ClinVar ID** | **Parent of Origin** |
| --- | --- | --- | --- | --- | --- | --- | --- | --- | --- |
| SLC6A5 | 11p15.1 | c.1429T>C | p.Ser477Pro | Missense variant | Substitution | not found | Compound heterozygous | SCV000897642 | Likely *de-novo* |
| SLC6A5 | 11p15.1 | c.1430delC | p.Ser477PhefsTer9 | Frameshift variant | Frameshift | rs767695215 | Compound heterozygous | SCV000897641 | From mother |

Supplementary Table S4 contains detailed coverage information on patient’s Mendelian sequencing panel. Supplementary Tables S5, S6, and S7 contain information on exome sequencing coverage for mother, father, and patient respectively. Each table shows minimum, maximum and average sequencing coverage for each exon along its number, chromosomal location, corresponding transcript, and gene.

**Supplementary Tables S8-10**

Supplementary Tables S8, S9, and S10 contain *de-novo* CNVs called by CoNIFER, XHMM, and ExomeDepth respectively.

**Supplementary Table S11**

**Supplementary Table S11:** Chromosome, HGVS DNA and protein references, variant types, predicted effects, and parent of origin for the *SLC6A5* variants, including dbSNP and ClinVar IDs.

**Supplementary Video S1:** Two separate instances of attacks of hyperekplexia in our patient, both at the age of two months.

**Supplementary Figure S1:**

**
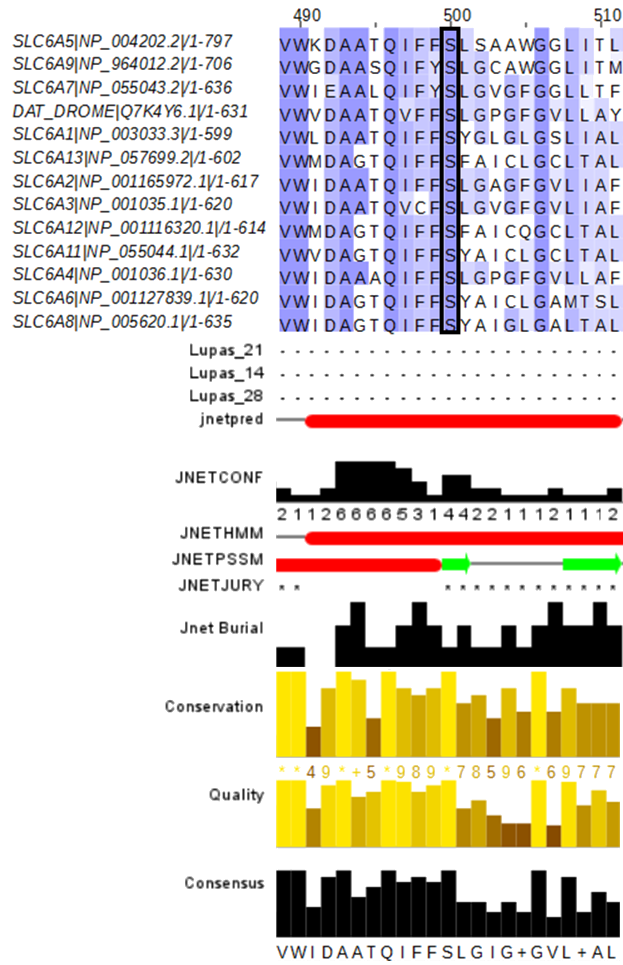
**

**Supplementary Figure S1:** Multiple sequence alignment of human SLC6A5 between the sodium-dependent dopamine transporter from Drosophila melanogaster (DAT_DROME) and members of the human SLC6 family indicates that the residue p.Ser477 is strictly conserved through sodium- and chloride dependent neurotransmitter transporters, highlighting its importance in sodium binding. Amino acid color labels were selected for the block substitution matrix 62. Overall secondary structure prediction (jnetpred) demonstrates the location of p.Ser477 in an α-helical section (red tube), consistent with its transmembrane location. As expected, no coiled-coil structure was predicted for this section (Lupas 21,14, 28). Prediction of solvent accessibility (Jnet Burial) shows a medium exposure of residue p.Ser477.

**References**

Abecasis GR, Auton A, Brooks LD, DePristo MA, Durbin RM, Handsaker RE, Kang HM, Marth GT, McVean GA. 2012. An integrated map of genetic variation from 1,092 human genomes. *Nature* **491**(7422): 56-65.

Adzhubei IA, Schmidt S, Peshkin L, Ramensky VE, Gerasimova A, Bork P, Kondrashov AS, Sunyaev SR. 2010. A method and server for predicting damaging missense mutations. *Nature methods* **7**(4): 248-249.

Alkan C, Coe BP, Eichler EE. 2011. Genome structural variation discovery and genotyping. *Nature reviews Genetics* **12**(5): 363-376.

Davydov EV, Goode DL, Sirota M, Cooper GM, Sidow A, Batzoglou S. 2010. Identifying a high fraction of the human genome to be under selective constraint using GERP++. *PLoS computational biology* **6**(12): e1001025.

Escaramis G, Docampo E, Rabionet R. 2015. A decade of structural variants: description, history and methods to detect structural variation. *Briefings in functional genomics* **14**(5): 305-314.

Flicek P, Amode MR, Barrell D, Beal K, Billis K, Brent S, Carvalho-Silva D, Clapham P, Coates G, Fitzgerald S et al. 2014. Ensembl 2014. *Nucleic acids research* **42**(Database issue): D749-755.

Fromer M, Moran JL, Chambert K, Banks E, Bergen SE, Ruderfer DM, Handsaker RE, McCarroll SA, O'Donovan MC, Owen MJ et al. 2012. Discovery and statistical genotyping of copy-number variation from whole-exome sequencing depth. *American journal of human genetics* **91**(4): 597-607.

Hong CS, Singh LN, Mullikin JC, Biesecker LG. 2016. Assessing the reproducibility of exome copy number variations predictions. *Genome medicine* **8**(1): 82.

Kawalia A, Motameny S, Wonczak S, Thiele H, Nieroda L, Jabbari K, Borowski S, Sinha V, Gunia W, Lang U et al. 2015. Leveraging the power of high performance computing for next generation sequencing data analysis: tricks and twists from a high throughput exome workflow. *PloS one* **10**(5): e0126321.

Krumm N, Sudmant PH, Ko A, O'Roak BJ, Malig M, Coe BP, Quinlan AR, Nickerson DA, Eichler EE. 2012. Copy number variation detection and genotyping from exome sequence data. *Genome research* **22**(8): 1525-1532.

Kumar P, Henikoff S, Ng PC. 2009. Predicting the effects of coding non-synonymous variants on protein function using the SIFT algorithm. *Nature protocols* **4**(7): 1073-1081.

Lappalainen I, Lopez J, Skipper L, Hefferon T, Spalding JD, Garner J, Chen C, Maguire M, Corbett M, Zhou G et al. 2013. DbVar and DGVa: public archives for genomic structural variation. *Nucleic acids research* **41**(Database issue): D936-941.

Li H, Durbin R. 2009. Fast and accurate short read alignment with Burrows-Wheeler transform. *Bioinformatics (Oxford, England)* **25**(14): 1754-1760.

Li H, Handsaker B, Wysoker A, Fennell T, Ruan J, Homer N, Marth G, Abecasis G, Durbin R. 2009. The Sequence Alignment/Map format and SAMtools. *Bioinformatics (Oxford, England)* **25**(16): 2078-2079.

McKenna A, Hanna M, Banks E, Sivachenko A, Cibulskis K, Kernytsky A, Garimella K, Altshuler D, Gabriel S, Daly M et al. 2010. The Genome Analysis Toolkit: a MapReduce framework for analyzing next-generation DNA sequencing data. *Genome research* **20**(9): 1297-1303.

Petrovski S, Wang Q, Heinzen EL, Allen AS, Goldstein DB. 2013. Genic intolerance to functional variation and the interpretation of personal genomes. *PLoS genetics* **9**(8): e1003709.

Ramu A, Noordam MJ, Schwartz RS, Wuster A, Hurles ME, Cartwright RA, Conrad DF. 2013. DeNovoGear: de novo indel and point mutation discovery and phasing. *Nature methods* **10**(10): 985-987.

Sherry ST, Ward MH, Kholodov M, Baker J, Phan L, Smigielski EM, Sirotkin K. 2001. dbSNP: the NCBI database of genetic variation. *Nucleic acids research* **29**(1): 308-311.

Stenson PD, Ball EV, Howells K, Phillips AD, Mort M, Cooper DN. 2009. The Human Gene Mutation Database: providing a comprehensive central mutation database for molecular diagnostics and personalized genomics. *Human genomics* **4**(2): 69-72.

Tan R, Wang Y, Kleinstein SE, Liu Y, Zhu X, Guo H, Jiang Q, Allen AS, Zhu M. 2014. An evaluation of copy number variation detection tools from whole-exome sequencing data. *Human mutation* **35**(7): 899-907.

Yeo G, Burge CB. 2004. Maximum entropy modeling of short sequence motifs with applications to RNA splicing signals. *Journal of computational biology : a journal of computational molecular cell biology* **11**(2-3): 377-394.
